# Effects of a Green Oat Herb Extract on Cognitive Performance and Neurophysiological Activity: A Randomized Double-Blind Placebo-Controlled Study

**DOI:** 10.3389/fnins.2021.748188

**Published:** 2021-10-01

**Authors:** Saul Martinez-Horta, Eran Ivanir, Tania Perrinjaquet-Moccetti, Matthias Heinrich Keuter, Jaime Kulisevsky

**Affiliations:** ^1^Movement Disorders Unit, Neurology Department, Hospital de la Santa Creu i Sant Pau, Barcelona, Spain; ^2^Biomedical Research Institute (IIB-Sant Pau), Barcelona, Spain; ^3^Centro de Investigación en Red-Enfermedades Neurodegenerativas (CIBERNED), Madrid, Spain; ^4^Frutarom Ltd., Migdal Haemeq, Israel; ^5^Frutarom Switzerland Ltd., Wädenswil, Switzerland

**Keywords:** cognition, ERP, avena, green-oat herb, p300, ERN, EEG, N2

## Abstract

Green oat extracts have been used for centuries in traditional medicine in view of their supposed beneficial effects on cognition and mood. Recently, a specific green oat formulation (Neuravena^®^) showed to have significant bioactive compounds potentially associated with the enhancement of processing speed, working memory and attention. The main aim of the current study was to compare the potential effect of acute administration of 800 mg of Neuravena^®^ with placebo on a set of neurophysiological correlates of processing speed, attention, performance-monitoring and inhibitory control. Twenty healthy participants were randomized to receive either Neuravena^®^ or placebo. Electroencephalographic (EEG) signal acquisition was obtained while participants carried out the modified Eriksen flanker and oddball tasks. Both groups were compared on measures of behavioral task performance, and a set of event-related potentials (ERPs) components related to performance monitoring (the error-related negativity; ERN and the N2), target detection, and attention (P3a/P3b). Following active-intervention N2, ERN, and P3a/P3b were significantly reduced and performance was faster, with no loss of accuracy. Conversely, no neurophysiological differences were found in the placebo group before and after treatment and performance worsened significantly in terms of reaction time and accuracy. Acute administration of 800 mg of Neuravena^®^ appears to enhance the optimization of neural resources and positively influences cognitive performance in tasks associated with executive functions, processing speed and attention. Moreover, Neuravena^®^ prevents the deleterious effects of tiredness during task performance.

## Introduction

Various forms and preparations of oat (*Avena sativa* L.) – also commonly known by names such as green oat and common oat - have been used for centuries to treat physical and psychological conditions (Blumenthal et al., 2000; Abascal and Yarnell, 2004). However, as with many other formulations used in traditional medicine, scientific evidence on the specific effects of this herb and on its mechanisms of action is lacking.

Previously, however, several studies have reported that the ingestion of a specific green oat preparation in healthy subjects has acute positive effects on cognitive functions and neurophysiological parameters. Positive effects have been observed in measures of speed of performance (global), executive function, working and episodic memory, attention, and quantitative electroencephalography (qEEG) (Berry et al., 2011; Dimpfel et al., 2011; Kennedy et al., 2017). In all these studies, a specific oat extract (Neuravena^®^) was used. Neuravena^®^ is green oat herb extract containing a range of potentially bioactive constituents. In bio-assay guided approaches, it has been shown to have a significant inhibitory effect on the monoamine oxidase B (MAO-B) and phosphodiesterase 4 (PDE-4) enzymes (Moccetti et al., 2006). Based on this finding, it has been proposed that increased dopaminergic availability and cerebral vasodilation underlie its reported acute positive effects (Moccetti et al., 2006; Kennedy et al., 2017). Chronic intake of the extract has also shown to improve vasodilator function in peripheral and cerebral arteries (Wong et al., 2013), suggesting a broad effect on brain function.

Dopamine plays a cardinal role in movement, cognition, and motivation. Proper functioning of the structures conveyed along basal ganglia-thalamo-cortical dopaminergic pathways is crucial for the optimal performance of several cognitive processes ascribed to frontal-executive functions. These include action and conflict monitoring, inhibitory control, attention, target detection, and processing speed (Goldman-Rakic, 1998; Nieoullon, 2002; Remy and Samson, 2003; Grahn et al., 2009). Event-related brain potentials (ERPs) derived from EEG recordings enable the study of neurophysiological activity and behavioral parameters in relation to these cognitive processes (Luck, 2005). The P3a and P3b components obtained during auditory oddball paradigms selectively reflect the automatic orientation of attention to novel stimuli (frontal P3a), target detection, and contextual update of information (parietal P3b) (Sutton et al., 1965; Courchesne et al., 1975; Escera et al., 2000; Friedman et al., 2001; Polich, 2007). Dopaminergic activity has been strongly associated with both P3a and P3b. Specifically, it appears to play a cardinal role in the frontal-central P3a and related frontal functions such as working memory and focal attention, but it also plays a partial role in the amplitude of P3b (Courchesne et al., 1975; Comerchero and Polich, 1999; Polich, 2007; Grahn et al., 2009). Error-related negativity (ERN) is a negative deflection that occurs after error responses, and it is part of the action monitoring system (Holroyd and Coles, 2002; Yeung et al., 2004; Gehring et al., 2018). It has been the subject of intensive research and has been interpreted as a neural correlate of error detection, originated due to the phasic dopaminergic changes of the neurons projecting from the mesencephalon to the anterior cingulate cortex (ACC) (Gehring and Knight, 2000; Luu and Tucker, 2001). Similarly, in conflict priming, it has been suggested that the frontal-central N2 component reflects cognitive control (Kopp et al., 1996; Nieuwenhuis et al., 2003; Kok et al., 2004; Ramautar et al., 2006; Folstein and Van Petten, 2008). The morphology of these ERPs has been found to be sensitive to changes caused by several conditions affecting the central nervous system (CNS), and also to changes induced by pharmacological agents (Aron et al., 2003; Fitzgerald et al., 2005; Riba et al., 2005; Lopez-Gongora et al., 2015; Hedges et al., 2016; Rabella et al., 2016; Seer et al., 2017).

Considering the previously reported findings from studies addressing the effects of Neuravena^®^, and taking into account the proposed mechanisms of action of this compound, we aimed to examine the relationship between indices of attention, performance-monitoring and inhibitory control abilities, such as the P3a, P3b, ERN, and N2 amplitudes and cognitive performance, as measured by neuropsychological tests in healthy individuals.

## Materials and Methods

### Design

This study had a single-session, randomized, double-blind, placebo-controlled design with two parallel study arms: Active intervention or placebo. Assessments were performed at baseline and 60 min after administering either the active intervention or placebo. The total running time of the experimental session was the same for all participants and consisted of a 60 min session of neurophysiological acquisition and task performance (baseline), a 60 min resting period at the beginning of which the active intervention or placebo was administered, and a 60 min session of neurophysiological acquisition and task performance after active intervention or placebo. The experimental session was conducted in the facilities of the Laboratory of Cognitive Neurophysiology and Human Neuropsychopharmacology at the Biomedical Research Institute Sant Pau, in an isolated room specifically prepared for conducting neurophysiological studies. As per protocol, before the experimental session was conducted, all participants were interviewed regarding adherence to the instructions provided in relation to the evening meal and breakfast as well as the quality of sleep and rest during the previous night.

To explore the acute effects of the studied compound, the experimental session was conducted on a single day, in the morning. Participants were requested to have their standard evening meal on the previous day but to avoid alcohol. In order to get a relative homogeneity on evening meal, participants were provided by a set of recommendations the amounts to be consumed and the types of foods that were recommended or should preferably be avoided. At breakfast, calorie intake was restricted to 200 kcal, and participants were required to take their normal amount of coffee to prevent potential caffeine withdrawal effects. Similar to what was done with evening meal, a list with possible breakfasts to consume and avoid was provided to participants. After the baseline assessment, during the resting period, participants received a 200kcal snack followed by the intervention.

### Participants

Participants were pre-selected from a database of healthy volunteers. Inclusion criteria were males or females aged 20 to 50 years and able to freely decide to participate in the study by signing the proper informed consent. Exclusion criteria were any history of alcohol and/or other substance abuse, major psychiatric events that required pharmacological management, symptoms suggestive of cognitive impairment, traumatic brain injury with loss of consciousness or any other disease of the CNS (i.e., epilepsy, migraine), neurosurgical intervention in the CNS, systemic disease – compensated or not- (i.e., diabetes), use of any dietary supplement, food allergies, body mass index (BMI) >35 or <18, or being pregnant at the time of screening. From the pre-selected volunteers, twenty participants were included in the study and randomly allocated to receive the placebo or the active compound. Although the final sample was relatively small, the total number of trials during EEG/ERPs acquisition was in accordance with the recommendations regarding sample size for ERPs studies (Larson and Carbine, 2017). The study included two groups of ten participants matched for main sociodemographic variables (age, gender, years of education, and BMI, summarized in Table 1).

**TABLE 1:** Sociodemographic and baseline characteristics of the sample.

	Total (*n* = 20)	Placebo (*n* = 10)	Active intervention (*n* = 10)	*p*
		
	Mean ± SD (range)			
Age (years)	25.5 ± 3.6 (19–34)	26.0 ± 3.8 (23–34)	24.9 ± 3.4 (19–30)	0.510
Gender (f/m)	9/11	6/4	3/7	0.370
Education (years)	13.0 ± 2.1 (8–18)	13.0 ± 1.3 (12–15)	13.0 ± 2.7 (8–18)	0.999
Weight (kg)	68.3 ± 7.1 (58–80)	68.0 ± 6.4 (58–76)	68.5 ± 8.1 (58–80)	0.880
BMI (kg/m^2^)	22.7 ± 1.7 (20–27)	22.1 ± 1.3 (20–23)	23.2 ± 1.8 (21–27)	0.148

*P value is based on independent *t*-test for continuous variables and Pearson’s χ2 for categorical variables.*

### Interventions

Both the placebo and encapsulated active intervention were provided by Frutarom Switzerland Ltd. The active intervention consisted of an extract (Neuravena^®^) manufactured from the dried above-ground parts of a selected variety of Avena sativa L., treated with aqueous ethanol (30% m/m) solution, filtered according to a proprietary filtration process and spray-dried. Twenty-eight per cent (m/m) Maltodextrin Ph. Eur. was added as a carrier together with 2% (m/m) of Silica, colloidal anhydrous Ph. Eur. Characteristics of the extract are drug-to-extract- ratio (DER) 3.5:1, and a flavonoid content (calculated as isovitexin) of ≥0.3% (w/w). The dosage form consisted of two hard-gelatin capsules, each containing 400 mg of extract or maltodextrin as placebo for a total of 800 mg. The reason for choosing 800 mg in the current study relies on a previous study (Kennedy et al., 2017), where the results suggested that the optimal dose of Neuravena^®^ in relation to improving cognitive performance lies at this level.

### Assessments

#### Stop-Signal Task

We used a modified version of the Eriksen flanker task (Lopez-Gongora et al., 2015). Participants were required to respond to the orientation of a central green arrow (target) with two additional green arrows on either side. To do so, they were instructed to signal with the right hand after a right-pointed target arrow and with the left hand after a left-directed target arrow. The four surrounding arrows either primed the target response (compatible trials, 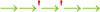 or 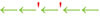) or primed the opposite direction (incompatible trials, 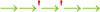 or 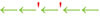). The task included 33% of compatible trials and 50% of incompatible trials. In the remaining 17% of trials we included “stop” trials. In these trials the central green arrow changed to red (for instance: 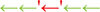) after a delay of 150ms and participants had to inhibit response in these trials. Duration of the stimuli was 300 ms. A random Stimulus-Onset Asynchrony (SOA) between 900 ms and 1100 ms was used. The experiment consisted of 3 blocks of 4 min, each with 200 stimuli. A 30-s rest period was allowed between blocks. Subjects were required to respond to the stimuli as fast and as accurate as possible and to inhibit their responses whenever a stop trial appeared. The percentage of total correct responses, and the reaction times (RT) to correct responses, and to error responses were recorded. Same measures were separately obtained for the compatible and incompatible trials.

#### Auditory Oddball Task

A standard auditory oddball task with a set of frequent, infrequent, and novel stimuli was used. Frequent stimuli (1500 Hz, 60 ms duration) occurred with a probability of 0.8, infrequent stimuli (1620 Hz; 60 ms duration) occurred with a probability of 0.1, and novel sounds (such as the sound of a key, or a door closing) occurred with a probability of 0.1 at 60 dB for 60 ms duration. Over a period of 15 min, participants were requested to respond as fast as possible to infrequent (target) sounds with the right index finger, and to ignore all other sounds. Percentage of correct responses to target and percentage or responses to non-target novel stimuli were recorded.

### Electroencephalogram Recording and Analysis

An electroencephalogram (EEG) was recorded using the BrainAmp system and Brain Vision Recorder Software (Brain Products GmbH, Germany) from 19 standard scalp sites according to the international 10/20 system (Fp1/2, F3/4, C3/4, T3/4, T5/6, P3/4, O1/2, F7/8, Fz, Cz, Pz) referenced to the two mastoid leads. Vertical eye movements were recorded using a bipolar montage with two electrodes linked together and placed below each eye referenced to a third electrode placed centrally above the eyes. Horizontal eye movements were monitored using two electrodes placed on the external canthi of each eye. Electrode impedances were kept below 5 kOhm. The electrophysiological signals were filtered with a bandpass of 0.1–35 Hz and digitized at a rate of 250 Hz.

To maximize the information available for the subsequent event-related potential analysis (ERPs), raw EEG signals were subjected to an ocular artifact minimization algorithm. This algorithm is based on an eigenvalue decomposition of time-delayed covariance matrices. After identifying the source signals associated with eye movements, we obtained corrected EEG signals from the remaining components. The algorithm was implemented using Brain Vision Analyzer (Brain Products GmbH; Germany).

#### P3a and P3b

In the auditory oddball task, the continuous EEG recording was segmented in epochs of 1000 ms starting 100 ms before the stimulus presentation and until 900 ms post-stimulus. Epochs were baseline-corrected, subtracting the mean amplitude in the 100 ms before the presentation of the stimulus. A two-step artifact rejection procedure was then used. First epochs were rejected if the signal in any of the 19 channels showed amplitude values greater than ± 300 μV. Subsequently, additional epochs were excluded if amplitude values were greater than ± 40 μV in Fz, Cz or Pz channels. After these preprocessing steps, three types of trials were averaged separately: epochs containing frequent stimuli, epochs containing infrequent stimuli, and epochs containing novel stimuli. These averages were obtained for each study participant and the ERP components were identified and quantified. The P3b was identified as the most positive deflection in the ERP between 300 and 700 ms post-stimulus in the infrequent trials. The P3a was identified as the most positive deflection in the ERP between 250 and 500 ms post-stimulus in the novel trials. The mean amplitude was calculated in the time-window defining each ERP and was introduced into the statistical analysis.

#### Error-Related Negativity and N2

In the stop task, ERPs were obtained following response and stimuli-locked average procedure. For the response-locked ERPs, the continuous EEG was segmented in epochs of 1000 ms, starting 200 ms before the commission of stop errors and before the correct responses until 800 ms thereafter. The 0-time point thus corresponded to a button press when the participant should withhold the response, and to the emission of a correct response. Baseline correction was performed subtracting the mean amplitude in the 100 ms before button press. Subsequently, we used the same two-step artifact rejection and procedure described above for P3a/P3b. For the stimulus-locked ERPs, we performed the same procedure as described for the acquisition and averaging of P3a or P3b, but on these occasions, we created two types of epochs: epochs containing trials compatible with correct responses and epochs containing trials incompatible with correct responses.

Following preprocessing, the epochs of the response-locked condition were averaged in order to obtain the ERN wave following commission errors. The ERN was identified as the negative deflection in the ERP appearing between 0 and 160 ms following a stop error. Quantification methods were used at Fz and Cz, because the ERN shows a frontocentral distribution. Accordingly, the average mean voltage was calculated between 0 and 160 ms following stop errors and correct responses. The N2 was identified as the negative deflection in the ERP appearing between 200 and 350 ms following the presentation of compatible and incompatible trials with correct responses. The mean amplitude was calculated at the defined time-window. As with the ERN, given the frontocentral distribution of the N2, it was quantified at Fz and Cz. The obtained average mean amplitudes for each subject were introduced into the statistical analysis described below. For depicting purposes, the signals obtained for each participant were averaged together providing a single grand average.

### Statistical Analysis

Sociodemographic and baseline characteristics were subjected to independent *t*-test comparisons between the two groups of interest for continuous variables and χ2 for categorical variables. Intervention effect on cognitive performance variable was analyzed using repeated-measures analyses of variance (ANOVA). Within each intervention arm, the effect was tested using paired *t*-test analysis. ERP effects were quantified for the three midline electrodes (Fz, Cz, and Pz), and the resulting data were subjected to repeated-measures ANOVAs with the Greenhouse-Geisser correction applied when necessary. *Post hoc* comparisons were performed using paired *t*-test comparisons. The statistical analysis was conducted using SPSS v25 software package.

## Results

Twenty healthy adults participated in this study (9 females and 11 males). Sociodemographic and baseline characteristics of the participants are shown in Table 1. Participants had a mean age of 25.5 years, mean weight of 68.3 and mean body mass index of 22.7 kg/m^2^. The two groups were matched for all the sociodemographic and baseline characteristics.

### Stop-Signal Task

*T*-test comparisons showed equivalent performance at baseline in the percentage of total correct responses [*t* = 0.179; *p* = 0.860)], in the percentage of correct responses in compatible trials [*t* = −0.377; *p* = 0.710] and in the percentage of correct responses incompatible trials [*t* = −0.178; *p* = 0.861]. Similarly, equivalent reaction times (RT) were found for all the conditions at baseline (*p* > 0.05; Table 2). Repeated measures ANOVA using the factors “intervention group” (placebo; active intervention) and “time” (baseline; post-intervention) showed a trend to interaction [*F* (1,18) = 3.71; *p* = 0.068] for the percentage of total correct responses, a trend to interaction [*F* (1,18) = 4.01; *p* = 0.060] for the RT to error responses, and a significant interaction for the RT to correct responses [*F* (1,18) = 11.80; *p* < 0.001], RT to compatible correct responses [*F* (1,18) = 5.09; *p* < 0.05], RT to incompatible correct responses [*F* (1,18) = 8.74; *p* < 0.01], and RT to incompatible error responses [*F* (1,18) = 4.78; *p* < 0.05] (Table 2).

**TABLE 2:** Behavioral performance in the Stop-signal task and in the Auditory oddball task at baseline and endpoint.

	Placebo		Active		*P* value	
	Baseline Mean ± SD	Change Mean ± SD	Baseline Mean ± SD	Change Mean ± SD	Baseline	Change
**Stop signal**						
**Total trials**						
Correct responses (%)	88.54 ± 5.96	−5.05 ± 9.31	89.08 ± 7.5	1.21 ± 4.21	0.860	0.068
RT in correct responses (ms)	357.02 ± 21.83	5.45 ± 12.2	376.84 ± 32.1	−17.72 ± 12.54^b^	0.124	<0.001
RT in errors (ms)	302.37 ± 24.41	11.67 ± 20.24	308.94 ± 13.40	−2.19 ± 8.33	0.465	0.060
**Compatible trials**						
Correct compatible responses (%)	95.13 ± 5.32	−2.54 ± 3.3	94.23 ± 5.33	0.44 ± 4.23	0.710	0.096
RT in correct responses (ms)	335.27 ± 15.59	2.33 ± 17.25	354.17 ± 33.13	−12.13 ± 2.33^b^	0.120	0.017
RT in errors (ms)	289.30 ± 44.06	14.13 ± 39.27	294.43 ± 21.17	1.13 ± 28.44	0.744	0.407
**Incompatible trials**						
Correct incompatible responses (%)	95.93 ± 4.26	−4.22 ± 6.33^a^	95.60 ± 4.01	−1.48 ± 5.34	0.861	0.309
RT in correct responses (ms)	370.17 ± 21.25	4.37 ± 20.68	393.82 ± 32.73	−18.21 ± 12.49^b^	0.071	0.008
RT in errors (ms)	305.19 ± 21.62	10.19 ± 18.55^a^	312.86 ± 13.87	−4.17 ± 9.32	0.360	0.042

**Auditory oddball task**						

Correct responses to target (%)	89.53 ± 9.84	−1.41 ± 6.51	88.27 ± 8.71	0.82 ± 4.23	0.765	0.375
Responses to novel stimuli (%)	3.23 ± 3.52	4.44 ± 8.68	4.12 ± 2.31	3.98 ± 7.52	0.512	0.900

*^*a*^ *p* ≤ 0.05 significance based on paired *t*-test (baseline and post-treatment in the placebo group)*

*^*b*^ *p* ≤ 0.05 significance based on paired *t*-test (baseline and post-treatment in the active group).*

**P* value: Based on two-sided *t*-test for independent samples (placebo vs. active).*

*Post hoc* paired *t*-test comparisons using the difference score between pre- and post-intervention administration showed that these effects were driven by significant decreases in several measures of RT in the active intervention group (Table 2). These effects were found for the RT to correct responses [*t* = −3.43; *p* < 0.005], the RT to compatible correct responses [*t* = −2.25; *p* < 0.05], and the RT to incompatible correct responses [*t* = −2.95; *p* < 0.01]. Effects were also seen in the placebo group in terms of increased RT to incompatible error responses [*t* = 2.18; *p* < 0.05) and in post-effects in terms of accuracy in the form of decreased performance “in the incompatible trials [*t* = −2.66; *p* < 0.05] (Table 2).

Bivariate correlation analysis showed significant negative effects after intervention in the placebo group (data not shown). Thus, the observed change in RT to incompatible error responses was significantly associated with worse performance in the placebo group in terms of percentage of correct responses (*r* = −0.458; *p* < 0.05).

**FIGURE 1:**
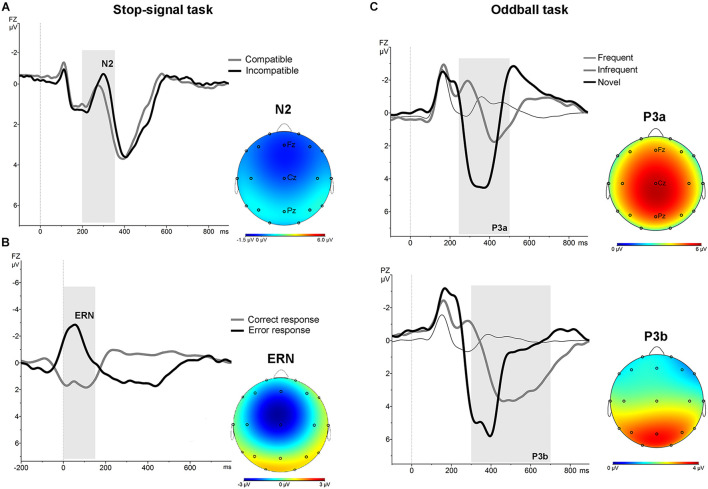
Stimulus-locked and response-locked (ERN) grand average ERPs for the whole sample in the stop-signal task. **(A)** Stimulus-locked grand average ERP at Fz electrode for compatible (grey line) and incompatible (black line) trials. The N2 is identified as the negative deflection in the ERP appearing between 200 and 350 ms (grey area). The topographical map shows the increased frontocentral negativity for incompatible trials. **(B)** Response-locked grand average ERPs at Fz electrode for correct (grey line) and error (black line) responses. The ERN is identified as the negative deflection in the ERP appearing between 0 and 160 ms (grey area). The topographical map shows the frontal-central ERN. **(C)** ERPs associated with frequent, infrequent and novel stimuli in auditory oddball task. Grand average stimulus-locked ERPs at Fz and Pz electrodes for the whole sample. The P3a was identified as the most positive deflection in the ERP between 250 and 500 ms (grey area) post-stimulus in the novel trials. The P3b was identified as the most positive deflection in the ERP between 300 and 700 ms post-stimulus in the infrequent trials. The topographical map shows the P3a and P3b.

In the stimulus-locked ERPs analysis, repeated measures ANOVA with the factors “electrode” (Fz and Cz) and condition (compatible/incompatible) showed a significant effect at Fz [*F* (1,19) = 19.8; *p* < 0.001] and Cz [*F* (1,19) = 13.52; *p* < 0.005] driven by a significant increased amplitude of the N2 at Fz [*t* = 4.45; *p* < 0.001], and Cz [*t* = 3.67; *p* < 0.005] in the signal averaged from the incompatible condition (Figure 1, part A). In the response-locked analysis, repeated measures ANOVA with the factors “electrode” (Fz and Cz) and “response” (correct, error) showed a significant effect at Fz [*F* (1,19) = 90.91; *p* < 0.001] and at Cz [*F* (1,19) = 133.45; *p* < 0.001], driven by a significantly increased amplitude at Fz [*t* = 7.96; *p* < 0.001] and at Cz [*t* = 9.54; *p* < 0.001] of the frontal-central negativity following an error response (ERN) (Figure 1, part B).

**FIGURE 2:**
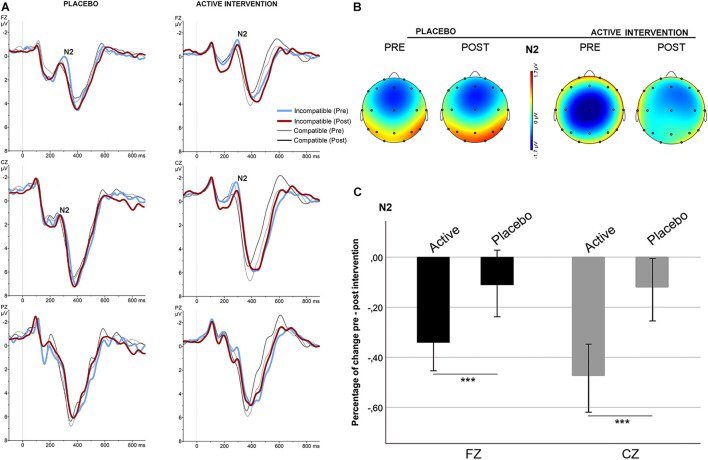
Stimulus-locked (grand average) ERPs for the N2 in the two groups. **(A)** ERPs after correct compatible (thin line) and incompatible (thick line) trials in each group, pre (blue/gray) and post (red/black) intervention. **(B)** The topographical map shows the pre-post effect on the mean amplitude of the N2 for incompatible condition. **(C)** Mean percentage of N2 change (in mV) for incompatible condition between study groups. *** significantly different (*p* < 0.05).

**FIGURE 3:**
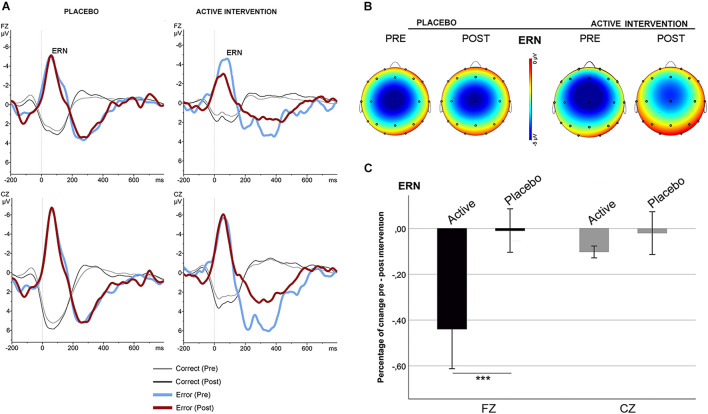
Response-locked (ERN) grand average ERPs after correct (thin line) and error (thick line) responses in the stop-signal task for each group, pre (blue/gray) and post (red/black) intervention. **(A)** ERPs comparison in the placebo and in the active intervention group. **(B)** The topographical map shows the pre-post effect on the mean amplitude of the ERN. **(C)** Mean percentage of ERN change (in mV) between study groups. *** significantly different (*p* < 0.05).

No significant differences were found at baseline between groups in the mean amplitude of N2 and ERN. Repeated measures ANOVA for the N2 using the factors “intervention group” (placebo; active intervention) and “time” (baseline; post-intervention) showed a significant group x time interaction at Fz [*F*(1,19) = 15.2; *p* < 0.001] and at Cz [*F*(1,19) = 19.3; <0.001], characterized by a decreased negativity of the N2 for the active-intervention arm (Figure 2). Bivariate correlation analysis showed that the reduction in the N2 amplitude was associated with an increase in the percentage of correct responses (*r* = −0.483; *p* < 0.05) and with a reduction in the RT to correct responses (*r* = 0.590; *p* < 0.01). Regarding the ERN, repeated measures ANOVA showed a significant group x time interaction at Fz [*F* (1,19) = 4.69; *p* < 0.05] (Figure 3). *Post hoc* comparisons showed that this effect was driven by a significant reduction of the ERN at Fz in the active-intervention arm after intervention [*t* = 2.23; *p* < 0.05].

### Auditory Oddball Task

**FIGURE 4:**
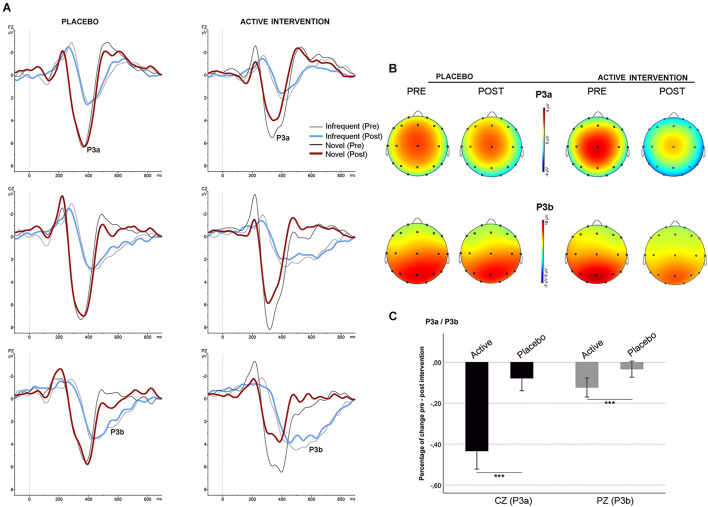
Stimulus-locked (grand average) ERPs for the P3a/P3b. **(A)** ERPs after infrequent (gray/blue line) and novel (black/red line) stimuli in each group. **(B)** Topographical maps showing the pre-post intervention effects on the P3a and P3b. **(C)** Mean percentage of P3a and P3b change (in mV) between study groups. *** significantly different (*p* < 0.05).

No differences were found between groups at baseline neither post-intervention in the percentage of correct responses to target and to non-target novel stimuli. Repeated measures ANOVA using the factors “electrode” (Fz, Cz, Pz) and “condition” (frequent/infrequent) showed significant effects at Cz [*F* (1,19) = 21.5; *p* < 0.001] and Pz [*F* (1,19) = 42.2; *p* < 0.001] driven by significant increased positivity at Cz [*t* = 4.63; *p* < 0.001] and at Pz [*t* = 6.5; *p* < 0.001] around 300 ms –700 ms following an infrequent stimulus (P3b). Similarly, adding novel stimuli to the factor “condition,” significant effects were found at Fz [*F* (1,19) = 53.4; *p* < 0.001], and Cz [*F* (1,19) = 81.5; *p* < 0.001] driven by a significantly increased positivity at Fz [*t* = 7.3; *p* < 0.001], and at Cz [*t* = 9; *p* < 0.001] around 250 ms – 500 ms following a novel stimulus (P3a) (Figure 1, part C). No significant differences were found between groups at baseline on the mean amplitude of the P3a and P3b components. Repeated measures ANOVA using the factors “intervention group” (placebo; active intervention) and “time” (baseline; post-intervention) showed a significant group x time interaction both for the P3a at Cz [*F* (1,19) = 3.30; *p* < 0.05] and for the P3b at Pz [*F* (1,19) = 4.68; *p* < 0.05]. *Post hoc* comparisons showed that these effects were driven by a significant reduction after the administration of intervention in the active-intervention arm of the P3a at Cz [*t* = 3.18; *p* < 0.05] and at Pz [*t* = 4.48; *p* < 0.005], and the P3b at Pz [*t* = 2.8; *p* < 0.05] (Figure 4). Bivariate correlation analysis showed that this reduction in the P3a component was significantly associated with the percentage of correct responses post-intervention in both compatible (*r* = −0.480; *p* < 0.05) and incompatible (*r* = −0.488; *p* < 0.05) trials (data not shown).

## Discussion

In the present study we explored the acute effects of ingestion of 800 mg of a green-oat herb extract (Neuravena^®^) on a set of behavioral and neurophysiological parameters. While previous studies have shown a potential modulatory effect of Neuravena^®^ on neurophysiological activity as measured by EEG in the fronto-temporal brain region, and successive improvements in several measures of cognitive function (Berry et al., 2011; Dimpfel et al., 2011; Kennedy et al., 2017), the current investigation provides evidence of an improvement in cognitive performance related to a concomitant neurophysiological effect. We specifically looked at potential effects on a set of neurophysiological signatures of action and conflict monitoring (ERN/N2), target detection, and attention (P3a/P3b). We also looked at the association between these neurophysiological measures and several behavioral variables.

Our results show that the two groups were strictly matched for the main variables of interest and that before the intervention there were no differences in any of the studied variables. However, in the active-intervention group, we found a set of post-intervention differences with respect to baseline and with respect to the placebo group in several neurophysiological and related behavioral parameters. Specifically, after active-intervention administration, we found significant reductions in several ERPs, and a significantly faster performance in several task components. Importantly, this faster performance was not associated with a worsening in accuracy. In contrast, in the placebo group, we found worsening effects both in RT and in accuracy.

We interpret the reduction in the mean amplitude of several ERPs as signatures of an acute effect of optimization of neural resources and cognitive performance in participants receiving the active intervention. These effects likely influence the maintenance of optimal performance in the task, as obvious signatures of tiredness were found in the placebo group.

In previous studies focusing on neurological disorders, compensatory mechanisms were observed during performance in the same tasks as those we used in the present study (Lopez-Gongora et al., 2015; Kropotov et al., 2016). Specifically, in ERP studies, the engagement of efficient compensatory mechanisms has been seen as a significant increase in ERP amplitude in association with the normalization of related behavior (Lopez-Gongora et al., 2015). This suggests that in these cases, participants use additional neural resources to perform tasks with no apparent difficulties. Similarly, throughout neurodevelopment, a progressive reduction in the amplitude of several ERPs is found in association with better performance in related tasks (Abdul Rahman et al., 2017). This progressive reduction may reflect progressive specialization along neurodevelopment of the neural resources needed to overcome task performance (Davis et al., 2003; Cragg and Nation, 2008; Abdul Rahman et al., 2017). Taken together, it is reasonable to assume in the present study that the reductions found in the ERPs in the group receiving the active intervention and the related positive behavioral consequences of these effects reflect better optimization of the neural resources required to perform the tasks, and better capacity to maintain optimal levels of performance, avoiding the tiredness effects found in the placebo group.

Taking into account the functional meaning of the studied ERPs and the related behavioral parameters, our results are consistent with previous data suggesting that Neuravena^®^ induces positive acute effects in processing speed and measures of attention and executive function (Berry et al., 2011; Dimpfel et al., 2011; Kennedy et al., 2017). More specifically, our data supports significant effects of Neuravena^®^ in terms of improving efficiency in action monitoring, cognitive control over interference and attention. Here, we demonstrate that in participants receiving a single dose of 800mg of Neuravena^®^, a reduction in the amplitude of the ERN is found with no deleterious effects on performance. Similarly, in the present study, participants in the active-intervention arm showed a reduction in the N2 component that was associated with faster performance both in compatible and incompatible trials. Specifically, incompatible trials are usually associated with greater RT than compatible trials. This greater RT is assumed to reflect the higher processing demands of the conflict monitoring system when participants are faced with incompatible trials. Thus, the significant reduction in RT in the active-intervention arm suggests increased efficiency of the conflict monitoring system to solve the processing demands driven by incompatibility. Conversely, increased latency of RT was found in the placebo group, affecting specific trials and thus suggesting a loss of performance efficiency along the experimental session.

During the auditory oddball task, both P3a and P3b were lower in the active-intervention group after intervention administration. The P3b is one of the most frequently studied ERPs in the history of cognitive neurophysiology. Although its specific functional meaning is only partially understood, it is clear that it is associated with processing speed and integration of information. Participants in the active-intervention group showed a significant decrease in the P3b component, with no deleterious effects on performance. Again, this finding supports the idea that more efficient neural resources are deployed by those using 800 mg of Neuravena^®^, promoting better performance. Equivalent findings were found for P3a component. The frontal P3a is specifically associated with automatic orientation of attention to novelty. Here, participants in the active-intervention group showed a significant reduction in the P3a component in association with a better performance in terms of accuracy. Again, more efficient neural resources and performance appear to be enhanced after the administration of 800 mg of Neuravena^®^.

The main strengths of the present study are the inclusion of a carefully matched sample of healthy individuals in each study arm and the use of several behavioral and neurophysiological metrics to assess the effects pre and post intervention. The study is not free of limitations that must be taken into consideration. The sample size is relatively small and does not allow large extrapolations of the results. Despite this, taking into account these results and the results obtained in previous studies, the current findings add evidence about the pro-cognitive effects of the studied compound. In this sense, although it is true that a larger sample would have favored the extrapolation of these findings, the effects observed in the different measures can be considered consistent. Unquestionably, however, more specific studies and clinical trials using larger samples will be necessary to help us to better understand the mechanisms involved in the reported findings and to allow the extrapolation of the results. Another limitation is that the sample size does not allow us to explore the possible association between the effects found and specific sociodemographic and baseline characteristics that may contribute to the magnitude of the effects. We also consider a limitation to have not collected some data or to have controlled for other variables that could play a role in the observed results. Among these, we highlight that the type and amount of intake made the day before and during breakfast could have been better controlled. These data were partially controlled by means of a previous interview but in future studies they should be objectified more specifically. The effect of fatigue throughout the two consecutive sessions could also play a role that must be taken into account. Similarly, the effects of inter and intraindividual variability in the ERPs between sessions could also play a role in the observed effects. In any case, since it is a study where the two groups were subjected to exactly the same experiment, we can consider that to a large extent, the effects mediated by fatigue and/or inter and intraindividual variability should be similar in both groups and that, therefore, the differences found would not be explained as a consequence of this factors. Finally, taking into account that the pro-cognitive effect of Neuravena^®^ constitutes the central element that have been studied in the present study, future studies should implement comprehensive neuropsychological assessment or additional tasks to more exhaustively address the influence of Neuravena^®^ over cognitive performance. Overall, some of the conclusions derived from the present study may be considered preliminary and in need of confirmation in future larger studies, but the set of results shed light on the potential effects of Neuravena^®^ in certain aspects of cognitive functioning in healthy individuals.

In summary, we found that a single dose of 800mg of the special green-oat herb preparation Neuravena^®^ was associated with acute modulations on several neurophysiological parameters associated with action monitoring, cognitive control, attention, and processing speed, and no negative effects were seen. The exact mechanisms subserving these effects are not totally understood. However, the suggested and demonstrated MAO-B inhibitory activity of Neuravena^®^ may play an essential role in these findings.

## Conclusion

Neuravena^®^ is associated with the optimization of neural resources and with increased efficiency in terms of performance and brain activity in healthy individuals. Further studies could help elucidate the effects of chronic administration of Neuravena^®^ on cognitive functions in health and in disease population.

## Data Availability Statement

The data that support the findings of this study are available on request from the corresponding author.

## Ethics Statement

The studies involving human participants were reviewed and approved by Ethics Review Board at Sant Pau Hospital. The patients/participants provided their written informed consent to participate in this study.

## Author Contributions

SM-H, TP-M, and JK: conception and design of the study. SM-H: recruitment of participants and assessments. SM-H, TP-M, and EI: data analysis. SM-H, EI, TP-M, MHK, and JK: interpretation, draft manuscript, and review. All authors contributed to the article and approved the submitted version.

## Conflict of Interest

EI and TP-M are employed by Frutarom Ltd. and Frutarom Ltd. Swizerland, respectively. MHK was employed by Frutarom Ltd. Swizerland. The remaining authors declare that the research was conducted in the absence of any commercial or financial relationships that could be construed as a potential conflict of interest.

## Publisher’s Note

All claims expressed in this article are solely those of the authors and do not necessarily represent those of their affiliated organizations, or those of the publisher, the editors and the reviewers. Any product that may be evaluated in this article, or claim that may be made by its manufacturer, is not guaranteed or endorsed by the publisher.
